# Safe and equitable medical radiation in the Asia–Pacific

**DOI:** 10.1016/j.lanwpc.2026.101918

**Published:** 2026-06-30

**Authors:** The Lancet Regional Health – Western Pacific

The seventy-ninth World Health Assembly (May 2026; Geneva, Switzerland) approved the first-ever resolution on radiation and health. This resolution recognises the increasing use of radiopharmaceuticals in medical diagnostics and treatment and underscores the need to ensure the safety and quality of medical uses of radiation. It is a call to build governance systems that determine when, where, and for whom these technologies deliver value.

The Asia–Pacific region shows a clear pattern of increasing use of medical radiation, accompanied by substantial regional disparities in accessibility and quality. Although high-income countries such as Japan, Australia, and South Korea offer comprehensive and advanced medical radiation services such as nuclear medicine, some settings still do not have basic access to quality diagnostic imaging and essential radiotherapy, especially in low-income countries, which creates avoidable delays, travel burdens, and worse health outcomes. The Philippines has only 112 radiation oncologists nationwide, and more than half of Filipinos must travel at least 100 km to access their nearest radiotherapy facility.

Widespread overuse of medical imaging coexists with the unmet need for diagnostic radiation in the region. With most evidence derived from high-income countries, overuse in other settings has not been well characterised. Rates of redundant repeated imaging continue to rise and a large proportion of imaging performed does not meet guideline criteria. This shortfall reflects uneven quality control and poor adherence to guidelines. For example, the underuse of clinical prediction rules, which can help rule out a possible disease before definitive testing, contributes to unnecessary imaging. Financial incentives, physicians’ fears of missing diagnoses, and poor public awareness of overdiagnosis also drive overuse. Inadequate staffing in the region further undermines the oversight of imaging services; some radiology departments in low-resource settings even function without any medical physicists. Unnecessary or poorly justified medical radiation exposes patients to avoidable harm, raises costs, and diverts scarce resources from people who truly need care.

Approaches to ensure safe and equitable medical radiation in the region can differ depending on population size, disease burden, geography, workforce, and health-system capacity but should be built around the same core aims—access, appropriateness, safety, and equity. Countries with limited capacity require investment in equipment, while innovative and resource-sparing methods of delivering radiotherapy are more feasible in settings with small populations and low caseloads. In countries where the workforce and supporting systems lag behind infrastructure capacity, the training of radiation oncologists, medical physicists, radiographers, and nuclear medicine specialists is indispensable.

Several interventions developed to address imaging overuse are being evaluated in the Asia–Pacific region. Audit-and-feedback strategies have shown modest but meaningful reductions in unnecessary imaging prescription, such as musculoskeletal imaging prescribed by general practitioners in Australia. Radiological clinical decision-support systems are promising but are not uniformly successful across the region, as it is as much an organisational and cultural process as a technological one. Establishment and implementation of quality and protocol governance, safety oversight, data-sharing systems, and equipment maintenance practices are essential to prevent potential adverse outcomes from harmful radiation exposure.

Maximising the benefits of medical radiation requires more than reducing unnecessary scans. Advanced technological innovations are promising to reshape the use of medical radiation. Artificial intelligence (AI) adoption and integration can help preserve image detail, reduce noise, and improve visual quality, while also optimising radiation dose and workflow. AI-based risk stratification models can reduce overuse of diagnostic radiation by improving patient selection and identifying groups at high risk who are most likely to benefit from radiation-based tests. For radiotherapy, a deep learning-enabled, fully automated quality assurance tool can assist clinicians in verifying accurate radiation dose targeting for palliative spine treatment planning, helping to prevent substantial treatment errors and improve the safety of spine radiotherapy. Although AI applications have the potential to improve medical radiation planning, it is important to carefully address issues related to data quality, model interpretability, and ethical considerations.

How to expand access to life-saving diagnostic imaging and radiotherapy while reducing unnecessary radiation exposure and unsafe use remains a core issue in our diverse region. There is a need for a regionally adapted model that can govern safe and equitable medical radiation across various health systems, with the goal of ensuring that every patient receives the right radiation-based test or treatment, in the right place, at the right dose, and for the right reason.

